# Human milk oligosaccharides promote immune tolerance via direct interactions with human dendritic cells

**DOI:** 10.1002/eji.201847971

**Published:** 2019-04-04

**Authors:** Ling Xiao, Wouter RPH van De Worp, Roderick Stassen, Celine van Maastrigt, Nienke Kettelarij, Bernd Stahl, Bernadet Blijenberg, Saskia A. Overbeek, Gert Folkerts, Johan Garssen, Belinda van't Land

**Affiliations:** ^1^ Utrecht Institute for Pharmaceutical Sciences (UIPS) Utrecht University Utrecht The Netherlands; ^2^ Department of Respiratory Medicine NUTRIM Maastricht University Medical Center Maastricht The Netherlands; ^3^ Departments of Immunology and of Human Milk Research & Analytical Science Danone Nutricia Research Utrecht The Netherlands; ^4^ Laboratory of Translational Immunology Wilhelmina Children's Hospital University Medical Center Utrecht Utrecht The Netherlands

**Keywords:** DC‐SIGN, human milk oligosaccharides, IL‐10, regulatory T cells (Treg), TLR4

## Abstract

Human milk oligosaccharides (HMOS) are a complex mixture of bioactive components supporting the immune development of breastfed‐infants. Dendritic cells (DCs) play a central role in the regulation of immune responses, being specialized in antigen presentation and driving T‐cell priming as well as differentiation. However, little is known about the direct effects of HMOS on human DC phenotypes and functions. Here, we report that HMOS mixture isolated from pooled human milk, induced semi‐maturation of human monocytes‐derived DCs (moDCs), and elevated levels of IL‐10, IL‐27 and IL‐6 but not IL‐12p70 and TNF‐α. Consistently, HMOS‐conditioned human moDCs promoted Treg generation from naïve CD4^+^ T cells. Interestingly, HMOS limited LPS‐induced maturation of human moDCs, while maintained IL‐10 and IL‐27 secretion and reduced LPS‐induced production of IL‐12p70, IL‐6 and TNF‐α. Furthermore, HMOS+LPS‐stimulated DCs induced a higher frequency of Tregs and increased IL‐10 production, while a reduction in Tbet+Th1 frequency and IFN‐γ production was detected as compared to LPS‐DCs. The regulatory effects of HMOS seemed to be mediated by interactions of HMOS with receptors, including but not limited to TLR4 and DC‐SIGN on human moDCs. In conclusion, HMOS contain tolerogenic factors influencing human moDCs and thereby modulating the development of the neonatal immune system.

## Introduction

In the past decades, there has been a growing appreciation that breast milk beneficially supports the innate immune system 1 and modulates microbiota composition during the first months of life. Breastfeeding is known to reduce the risk of inflammatory diseases 2, infectious diseases 3, 4, asthma and allergic disease 5, as well as autoimmune diseases 6. Among the various bioactive components in breast milk, human milk oligosaccharides (HMOS) have been suggested to play a key role 7, 8. HMOS are a diverse mixture of over 1000 individual oligosaccharides that belong to different core structures consisting of short chain and long chain oligosaccharides typically present in a 9:1 ratio. In this paper the term “HMOS” is used as term for the complete mixture of HMOS, when individual compounds are referred to, they are labelled with their specific name. Among the most abundant oligosaccharides present in the majority of human milk samples, are 2’‐fucosyllactose (2’FL), 3’‐sialyllactose (3’SL), 6’‐sialyllactose (6’SL), and Lacto‐N‐Fucopentaose I (LNFP‐I) 9, 10, with a disaccharide lactose backbone at the reducing end, consisting of glucose, galactose, N‐acetyl‐glucosamine, fucose and sialic acid 7, 11. Despite facilitating the outgrowth of specific bacteria by serving as metabolic substrates, thereby beneficially shaping the microbiota composition and metabolism 12, HMOS may also directly modulate the immune system. It is known that HMOS are sampled by the lamina propria residing dendritic cells (DCs) 13. In addition, some structures have been detected in systemic circulation, suggesting absorption in the small intestine 14, 15. These findings indicate possibilities of direct interaction between HMOS and human DCs.

DCs are pivotal in the regulation and development of innate and adaptive immune responses during infections and inflammatory diseases. Upon sensing environmental signals, DCs mature and adjust their cytokine microenvironment; they migrate from the peripheral tissues into and within secondary lymphoid organs to induce and regulate effector T cell responses. One sub‐population of DCs, the so‐called tolerogenic dendritic cell (tDC) 16, 17, 18, are featured by lower levels of co‐stimulatory molecules such as CD80, CD86, CD40, but high expression of inhibitory markers such as PD‐L1 and OX40L. These tDCs respond with a reduced inflammatory cytokine production such as IL‐12, IL‐4, IL‐6, and TNF‐α, but elevate the regulatory cytokine production including IL‐10, TGF‐β, and IL‐27. More importantly, tDCs are functional regulatory T cell (Treg) inducers. Tregs exert their immunosuppressive capacity through a variety of mechanisms affecting again both DCs and effector T‐cell 19. Since the neonatal immune system is fully in development and can be characterized by an immature Th1 immune responsiveness and predominant Th2 activity 20, the induction of tDCs can be seen as a potential target for prevention and treatment of autoimmune diabetes 21 and allergic diseases 16 early in life.

The induction of tDCs has been experimentally achieved by tolerogenic factors such as prebiotic oligosaccharides and some commercially available individual HMOS. In human monocytes‐derived DCs (moDCs), a mixture of short‐chain galacto‐oligosaccharides (scGOS) and long‐chain fructo‐oligosaccharides (lcFOS) at a ratio of 9:1, directly modulated DCs phenotype. Both cytokine and chemokine production and T‐cell priming were modulated in a TLR4‐mediated manner in vitro 22. In a murine colitis model pro‐inflammatory stimulation was induced by 3’SL, possibly through direct recognition by intestinal DCs in a TLR4‐dependent manner, whereas 6’SL was not able alter the DCs 23. Due to the immature immune system of neonates, it can be suggested that specific milk oligosaccharides may provide structural cues to educate the early innate immune system and prepare the infant for a possible encounter with pathogenic bacteria.

Despite advances within this field, there is scarce data regarding possible direct immunomodulatory effects of the naturally occurring HMOS mixture on human DCs phenotype and subsequent DC‐T‐cell interaction. Consequently, the underlying mechanism through which HMOS may exert their immunomodulatory effects on human DCs, during a period of changing environmental factors, remains unclear. Several receptors through which specific HMOS structures may exert their immunomodulatory effects on DCs have been postulated. For example, the LPS recognizing TLR4 on DCs was suggested to be involved in the effects of 3’FL on DCs both in vitro 24 as well as in vivo 23. TLR4 has also been suggested to bind to LNFP III which subsequently promoted Th2‐like responses 25, although part may possible by induced by possible endotoxin contamination 24. In addition, DC‐specific intercellular adhesion molecule‐3‐grabbing non‐integrin (DC‐SIGN) has been suggested to be a major receptor of fucosylated HMOS specifically 26; and sialic acid binding immunoglobulin‐like lectins (Siglecs) have been shown to bind sialylated oligosaccharides from human milk 27. However, whether these postulated interactions are responsible for distinct biological effects of specific HMOS on DCs remain to be established.

Therefore, we set out to assess whether and through which molecular mechanisms HMOS may influence the phenotype and function of human moDCs. In addition, the influence of HMOS on LPS‐driven maturation and functionality of human moDCs was tested. The results indicate that HMOS can induce human tDCs, thereby supporting the immune developmental capacity of human milk. Identification of possible pathways involved in the induction of this regulatory phenotype by HMOS may provide new insight into the cellular and molecular mechanisms through which HMOS can exert their immunomodulatory effects in humans.

## Results

### HMOS induce semi‐maturation and attenuate LPS‐induced activation of human moDCs

**Figure 1:**
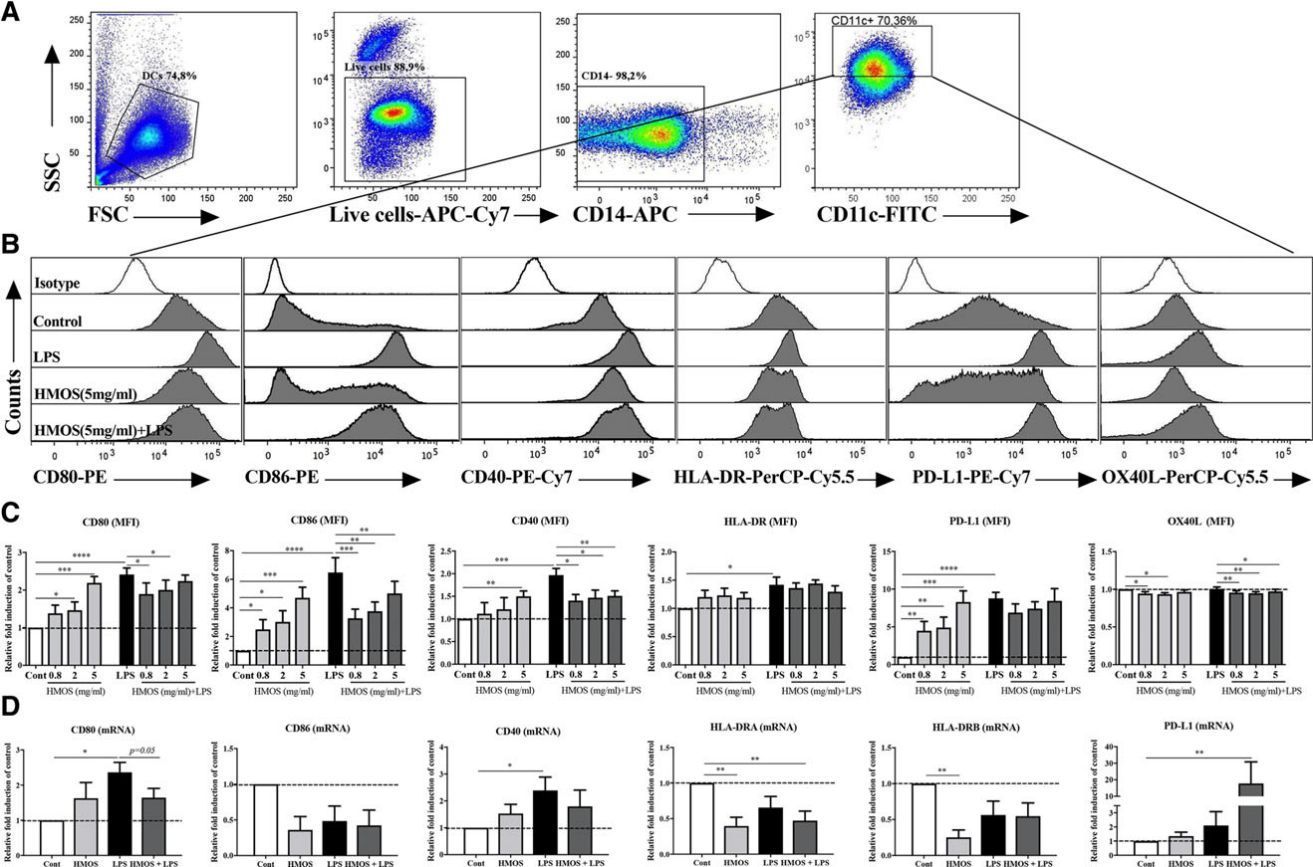
Effects of HMOS on the maturation status of human moDCs in the absence or presence of LPS. (A) Gating strategy of CD14‐APC negative DCs. (B) Representative histograms and (C) (Median Fluorescence Intensity (MFI)) of co‐stimulatory molecules CD80‐PE, CD86‐PE, CD40‐PE‐Cy7, and HLA‐DR‐PerCP‐Cy5.5, and inhibitory molecules PD‐L1‐PE‐Cy7 and OX40L‐PerCP‐Cy5.5. expression of DCs treated by medium, LPS, HMOS (0.8, 2, 5 mg/mL), and HMOS+LPS. Open histograms represent isotype mAb staining. The y‐axis of the column bar graphs shows the relative expression of surface markers obtained by setting medium control as 1‐fold within one experiment for each donor. (D) mRNA levels of co‐stimulatory molecules CD80, CD86, CD40, and HLA‐DRA, HLA‐DRB, and inhibitory molecule PD‐L1 expression of DCs treated by medium, LPS, HMOS (5mg/ml), and HMOS+LPS. The y‐axis of the column bar graphs shows the relative mRNA expression obtained by setting medium control as 1‐fold within one experiment for each donor. Immature DCs were generated from human PBMC‐derived monocytes, on day 6 immature DCs were stimulated with either medium (control), or HMOS (0.8, 2, 5 mg/mL) in the presence or absence of 100 ng/mL LPS. DCs were collected at 24 h for flow cytometry analysis or at 16 h for mRNA levels by qPCR analysis. Results are presented as mean ± SEM (7‐9 independent experiments (1‐2 donors/experiment) for flow cytometry analysis, and 2 independent experiments (2‐3 donors/experiment) for QPCR analysis). *p < 0.05, **p < 0.01, ***p < 0.001, ****p < 0.001, paired Student's *t*‐test.

The tolerogenic function of tDCs is determined by their activation status, they are often semi‐matured (reduced expression of surface markers like MHC‐II) with lack of pro‐inflammatory cytokines production but capable to interfere with activation induced by pro‐inflammatory signals like LPS 16. To test the effect of HMOS on the expression of MHC‐II and costimulatory molecules on human moDCs and during co‐stimulation with LPS, flow cytometry and qPCR analysis were conducted. Stimulation of immature DCs with LPS and/or HMOS at the highest concentration (5 mg/ml) did not affect the cell viability (Supporting Information Fig. 2). Within a physiological relevant concentration range from 0.8 to 5 mg/mL 9, 10, HMOS showed no significant impact on the protein expression of HLA‐DR (Fig. 1B, C), however upregulated the expression of co‐stimulatory molecules CD80 (Fig. 1B, C, *p* < 0.05 and *p* < 0.001 for HMOS at 2 and 5 mg/mL, respectively), CD86 (Fig. 1B, C, *p* < 0.05, *p* < 0.05, and *p* < 0.001 for HMOS at 0.8, 2 and 5 mg/mL, respectively), and CD40 (Fig. 1B, C, *p* < 0.01 for HMOS at 5 mg/mL) in a concentration‐dependent manner, but not to a full extent compared to the LPS‐induced increase of these surface molecules (Fig. 1B, C). In addition, HMOS upregulated the expression of inhibitory markers PD‐L1 (Fig. 1B, C, *p* < 0.01, *p* < 0.01, and *p* < 0.001 for HMOS at 0.8, 2 and 5 mg/mL, respectively), while down‐regulated the expression of OX40L (Fig. 1B, C, *p* < 0.05 and *p* < 0.05 for HMOS at 0.8 and 2 mg/mL, respectively) compared to untreated control DCs (gating strategy shown in Fig. 1A). At mRNA level, HMOS at 5 mg/mL did not significantly influence the expression of the surface markers except for a significant downregulation in HLA‐DRA (Fig. 1D, *p* < 0.01) and HLA‐DRB (Fig. 1D, *p* < 0.01). These data suggest that HMOS addition to immature PBMC derived human moDC induce semi‐maturation as compared to untreated control DCs and LPS‐induced fully matured DCs. LPS alone induced significant levels of CD80 (*p* < 0.0001), CD86 (*p* < 0.0001), CD40 (*p* < 0.001), and HLA‐DR (*p* < 0.05) compared to untreated DCs (Fig. 1B, C). With a co‐incubation of HMOS and LPS, the protein expression of CD80, CD86, and CD40 were suppressed significantly in a concentration‐dependent manner, whereas no change in expression of the inhibitory marker PD‐L1 was detected and expression of OX40L was decreased as compared to LPS alone (Fig. 1B, C). At mRNA level, LPS increased the mRNA expression of CD80 (Fig. 1D, *p* < 0.05) and CD40 (Fig. 1D, *p* < 0.05) compared to untreated control. Co‐incubation with the combination of HMOS and LPS slightly decreased the CD80 (Fig. 1D, *p* = 0.05) mRNA levels and significantly downregulated HLA‐DRA levels (Fig. 1D, *p* < 0.01), but significantly increased the mRNA expression of PD‐L1 (Fig. 1D, *p* < 0.01) compared to untreated control. These findings indicate that HMOS co‐incubation interferes with the LPS‐induced maturation of human moDCs.

Importantly, observed effects of HMOS seemed unlikely to be induced due to potential LPS contamination as analyzed using polymyxin B (PB) pretreatment experiments. In a control assay, PB, an antibiotic recognized for its LPS‐neutralizing effects, was added to LPS and HMOS (5 mg/mL) solution before treating the moDCs, and expression of CD80 and IL‐10 production were assessed. Addition of PB effectively abolished LPS‐induced upregulation of CD80 (Supporting Information Fig. 3A, *p* < 0.05, LPS versus LPS+PB) and IL‐10 production (Supporting Information Fig. 3B, *p* < 0.05, LPS versus LPS+PB) of DCs, indicating the neutralizing effects of PB on LPS in this human moDC model. However, no difference was observed in HMOS‐induced CD80 expression (Supporting Information Fig. 3A, no significance between HMOS and HMOS+PB) and IL‐10 production (Supporting Information Fig. 3B, no significance between HMOS and HMOS+PB) by adding PB. These results indicate that a potential LPS contamination within HMOS was not sufficient to induce HMOS‐related effects.

### HMOS induce an anti‐inflammatory cytokine profile in human moDCs

**Figure 2:**
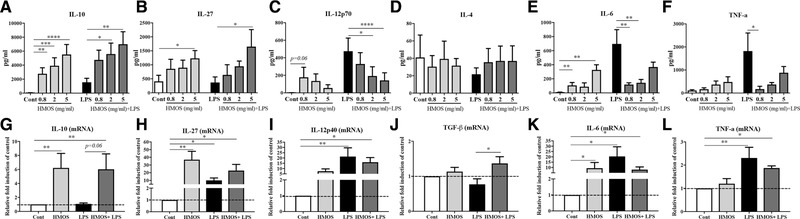
Effects of HMOS on cytokines release by human moDCs in the absence or presence of LPS. Production of (A) IL‐10, (B) IL‐27, (C) IL‐12p70, (D) IL‐4, (E) IL‐6, and (F) TNF‐α by DCs treated with medium, HMOS at 0.8, 2 and 5mg/mL in the presence or absence of 100 ng/mL LPS. mRNA expression of (G) IL‐10, (H) IL‐27, (I) IL‐12p40, (J) TGF‐β, (K) IL‐6, and (L) TNF‐α of DCs treated with medium, 5 mg/mL HMOS, LPS or 5 mg/mL HMOS+LPS. Immature DCs were stimulated with either medium (control), 100 ng/mL LPS (positive control), (0.8, 2 or 5 mg/mL) HMOS or HMOS in the presence of 100 ng/mL LPS. After 24 h, supernatants were collected and analyzed for IL‐10 (A), IL‐12p70 (B), IL‐6 (C), TNF‐a, and IL‐27 release by ELISA assays and cells were collected for QPCR analysis. Results are presented as mean ± SEM (3 independent experiments (2‐3 donors/experiment) for Elisa assays and 2 independent experiments (2‐3 donors/experiment) for QPCR analysis). For panel G‐L, the y‐axis of the column bar graphs shows the relative mRNA expression obtained by setting medium control as 1‐fold within one experiment and for each donor. *p < 0.05, **p < 0.01, ***p < 0.001, ****p < 0.0001, Paired Student's *t*‐test.

Only surface expression level changes on human moDCs are not sufficient to assess the DC function. Therefore, cytokine production was determined in supernatants of human moDCs treated with HMOS. An increase in the regulatory cytokine IL‐10 (Fig. 2A, *p* < 0.01, *p* < 0.001, and *p* < 0.001 for HMOS at 0.8, 2, and 5 mg/mL, respectively) and IL‐27 (Fig. 2B, *p* < 0.05 for HMOS at 5mg/mL) production was detected in the supernatant of HMOS‐conditioned DCs. Whereas no difference was detected in the Th1‐type of cytokine IL‐12p70 (Fig. 2C) and Th2‐type of cytokine IL‐4 (Fig. 2D) production between HMOS‐conditioned DCs and untreated control DCs. Surprisingly, an increase in the pro‐inflammatory cytokine IL‐6 was detected in the supernatant of HMOS‐conditioned DCs (Fig. 2E, *p* < 0.01 and *p* < 0.01 for HMOS at 0.8 and 5 mg/mL, respectively). No significant effect was detected in the pro‐inflammatory cytokine TNF‐α production of human moDCs by HMOS (Fig. 2E). Stimulation of human moDCs with LPS effectively elevated the release of IL‐10 (Fig. 2A, *p* < 0.001), IL‐12p70 (Fig. 2C, *p* < 0.0001), IL‐6 (Fig. 2E, *p* < 0.01) and TNF‐α (Fig. 2F, *p* = 0.05) levels in the supernatant of moDCs compared to untreated control, while no effect on the DCs production of IL‐27 (Fig. 2B) and IL‐4 (Fig. 2E) was detected by LPS stimulation. Co‐incubation of HMOS and LPS significantly increased levels of IL‐10 (Fig. 2A, *p* < 0.05 and *p* < 0.01 for HMOS at 2 and 5mg/mL, respectively) and IL‐27 (Fig. 2F, *p* < 0.05 for HMOS at 5 mg/mL) in the supernatant of moDCs as compared to control DCs. On the other hand, co‐incubation of HMOS and LPS completely suppressed the release of pro‐inflammatory cytokines IL‐12p70 (Fig. 2C, *p* < 0.05 and *p* < 0.0001 for HMOS at 2 and 5 mg/mL, respectively), IL‐6 (Fig. 2E, *p* < 0.01 and *p* < 0.01 for HMOS at 0.8 and 2 mg/mL, respectively), and TNF‐α (Fig. 2F, *p* < 0.05 for HMOS at 0.8 mg/mL) as compared to LPS treated DCs. Consistently, at mRNA level, an increased expression of IL‐10 (Fig. 2G, *p* < 0.01), IL‐27 (Fig. 2H, *p* < 0.01) and IL‐6 (Fig. 2K, *p* < 0.05) was detected in the DCs treated by HMOS (5mg/ml) as compared to control DCs. No differences in mRNA levels of IL‐12p40 (Fig. 2I) and TNF‐α (Fig. 2L) expression were observed between DCs with different treatments. Within DCs treated with HMOS+LPS, the mRNA levels of IL‐12p40 (Fig. 2I, *p* < 0.05) and IL‐6 (Fig. 2K, *p* < 0.05) expression was increased as compared to non‐treated DCs. Besides, an increased mRNA expression of TGF‐β was detected in the combination HMOS and LPS condition compared to LPS treatment alone (Fig. 2J, *p* < 0.05). Collectively, these results indicate that the addition of HMOS alone to moDCs results in cytokine production with a more regulatory phenotype, whereas the LPS‐induced maturation and (pro‐) inflammatory cytokine release by human moDCs, was inhibited by the co‐incubating with HMOS.

### HMOS induce the expression of migration markers in human moDCs

**Figure 3:**
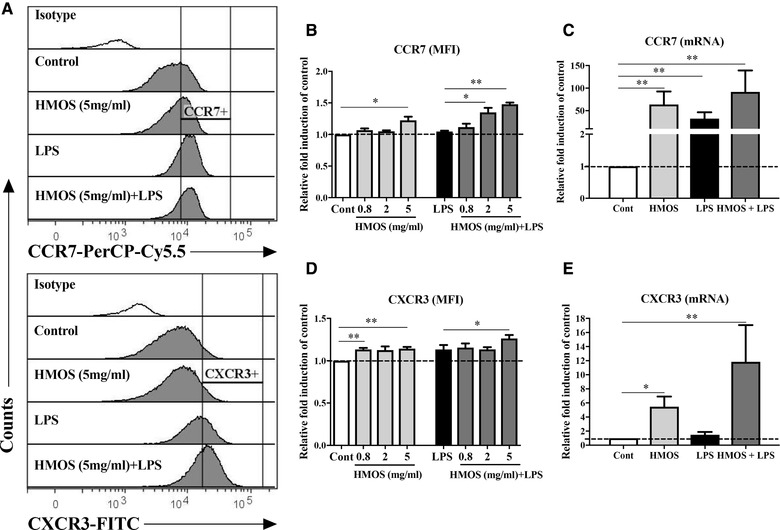
Effects of HMOS on the migratory marker's expression of human moDCs in the absence or presence of LPS. (A) Representative histograms of CCR7‐PerCP‐Cy5.5 and CXCR3‐FITC expression on the DCs treated by medium, LPS, HMOS (5 mg/ml), and HMOS (5 mg/ml) + LPS. Open histograms represent isotype mAb staining. MFI of (B) CCR7 and (D) CXCR3 expression on human DCs. mRNA expression of (C) CCR7 and (E) CXCR3 on human DCs. iDCs were generated and treated as described in the figure legend 1, after 24 h, DCs were analyzed by flow cytometry and qPCR for the expression of the migration markers CCR7 and CXCR3 at protein and mRNA levels, respectively. Results are presented as mean ± SEM (3 independent experiments (1–2 donors/experiment)). *p < 0.05, **p < 0.01, paired Student's *t*‐test.

Another feature of the previously described semi‐mature tolerogenic DCs is their lymph node or inflamed site homing potential, by which DCs can guide T‐cell to the proper anatomical locations. We therefore examined on HMOS‐conditioned DCs the expression of chemokine receptors CCR7 and CXCR3, which are known to enable DC's migration to lymphoid organs and inflammatory lesions respectively 27. An increased expression of CCR7 (Fig. 3B, *p* < 0.05; Fig. 3C, *p* < 0.01) and CXCR3 (Fig. 3D, *p* < 0.05; Fig. 3E, *p* < 0.01) at both the protein and mRNA levels respectively was detected on the moDCs pre‐incubated with HMOS (5 mg/mL), compared to untreated control DCs. LPS is essential for the induction of migratory activity in tolerogenic DCs 28. Upon LPS stimulation, CCR7 mRNA expression was increased (Fig. 3C, *p* < 0.01). Upon addition of both HMOS and LPS, an increased expression of CCR7 and CXCR3 at both protein and mRNA levels were detected compared to untreated control (Fig. 3B‐E). The increase in expression of CCR7 and CXCR3 suggests that HMOS may be able to equip human DCs with the capacity to migrate to lymphoid organs and inflammatory lesion, respectively. In addition, LPS stimulation seems to enhance this capacity.

### HMOS‐conditioned human moDCs support regulatory T‐cell development

**Figure 4:**
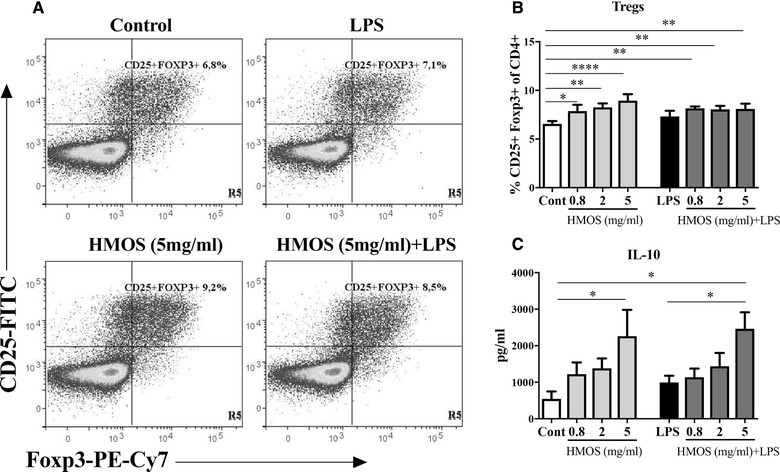
Effects of HMOS‐conditioned human moDCs on the induction of Tregs and IL‐10 production by T‐cell. (A) Representative plots of CD4^+^CD25+Foxp3+ Tregs induced by control DCs treated with medium, LPS, HMOS (5 mg/mL), and HMOS (5 mg/mL) +LPS matured DCs. Gating strategy is shown in Supporting Information Fig. 4. (B) Percentage of CD25+Foxp3+ Tregs from CD4^+^ T‐cell 6–7 days’ post MLR assay. Stimulated DCs were collected at 24 h and co‐cultured with fresh isolated naïve CD4^+^ T‐cell in a 1:10 ratio. After 6–7 days of co‐culture, T‐cell was harvested and analyzed for the expression of CD4‐PerCP‐Cy5.5, CD25‐FITC and Foxp3‐PE‐Cy7 by flow cytometry. (C) IL‐10 secretion in the supernatant of MLR. Supernatant was collected for IL‐10 measurement by ELISA assay. Results are presented as mean ± SEM (4 independent experiments (2‐3 donors/experiment) for panel B, (3 independent experiments (1‐2 donors/experiment) for panel C), *p < 0.05, **p < 0.01, ****p < 0.0001, paired Student's *t*‐test.

Both IL‐10 and IL‐27 released by moDC, are linked to the induction of CD4^+^ T‐cell with regulatory function 19, 21, 22. Conversely, IL‐12p70 has been shown to reduce the frequency of regulatory T‐cell and Foxp3 expression and enhance the activation and proliferation of conventional T‐cell 23. To examine whether HMOS‐conditioned DCs can induce regulatory T‐cell development, we conducted an allogenic mixed lymphocyte reaction (MLR) with the stimulated DCs and purified naïve CD4^+^ T‐cell from PBMCs. After 6 days of co‐culture, CD4^+^ T‐cell was phenotypically analyzed for the expression of CD25 and Foxp3. Compared to untreated moDCs, HMOS‐conditioned DCs induced up to 2.5% higher CD25+Foxp3+ Tregs within the CD4^+^ T‐cell population (Fig. 4B left panel, *p* < 0.05, *p* < 0.01 and *p* < 0.001 for 0.8, 2, and 5 mg/mL HMOS, respectively). No differences were detected on the percentage Tregs when DCs were stimulated by LPS compared to untreated DCs. In a culture of moDCs conditioned by the combination of LPS and HMOS (0.8, 2, and 5 mg/mL) up to 1.6% higher Tregs were detected compared to untreated control DCs (Fig. 4B right panel, *p* < 0.01, *p* < 0.01, *p* < 0.01 for 0.8, 2, and 5 mg/mL HMOS, respectively). Consistently, HMOS‐conditioned DCs significantly increased IL‐10 production by CD4^+^ T‐cell after 6‐day co‐culture (Fig. 4C left panel, *p* < 0.05 for HMOS at 5 mg/mL), while compared to control DCs, LPS stimulated DCs did not significantly influence the IL‐10 production by CD4^+^ T‐cell. In addition, a higher level of IL‐10 was detected in supernatants of T‐cell co‐cultured with DCs treated by LPS and 5 mg/mL HMOS, compared to LPS‐DCs and untreated control DCs (Fig. 4C right panel, *p* < 0.05, *p* < 0.05, respectively). Together, these findings confirmed the regulatory phenotype of HMOS‐conditioned human moDCs, and again, the presence of LPS did not abolish this phenotype.

### HMOS‐conditioned human moDCs influence Th1 differentiation

**Figure 5:**
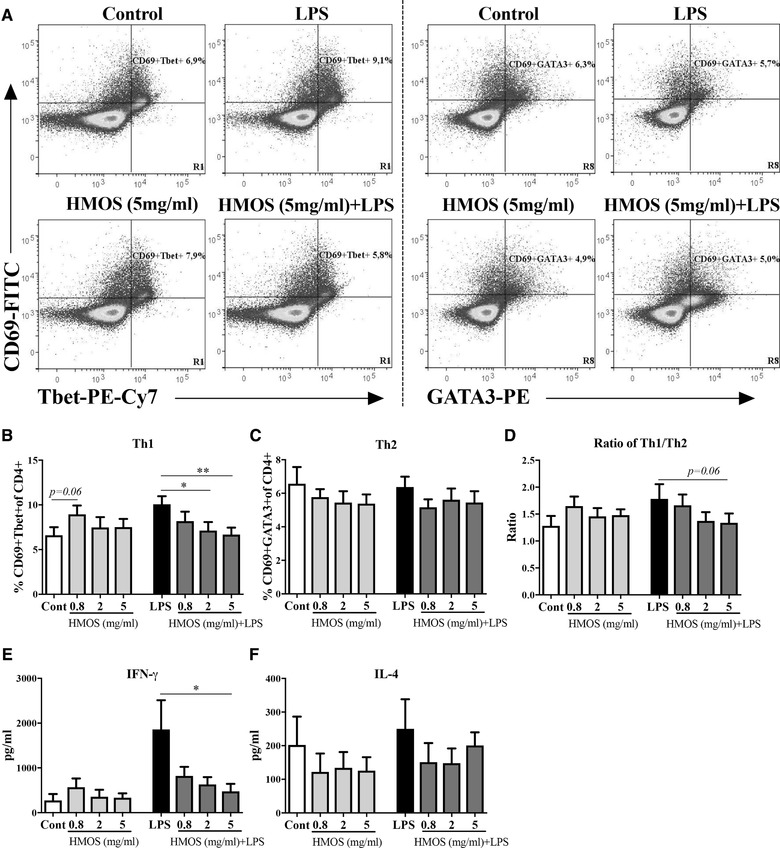
Effects of HMOS‐conditioned human moDCs on the prime of Th1 and Th2 responses. (A) Representative plots of CD69+Tbet+ (left panels) and CD69+GATA3+ (right panels) CD4 T‐cell. Gating strategy is shown in Supporting Information Fig. 4. Percentage of (B) CD69+Tbet+ (Th1) and (C) CD69+GATA3+ (Th2) cells of the CD4^+^ T‐cell population. (D) Ratio of Th1 and Th2 cells. Production of (E) IFN‐γ and (F) IL‐4 by co‐cultured T‐cell. The expression of CD4‐PerCp‐Cy5.5, CD69‐FITC, Tbet‐PE‐Cy7, and GATA3‐PE was analyzed by flow cytometry. Results are presented as mean ± SEM, (4 independent experiments (2‐3 donors/experiment) for panel B‐D, (3 independent experiments (1‐2 donors/experiment) for panel E,F). *p < 0.05, **p < 0.01, paired Student's *t*‐test.

To examine the effects of HMOS‐conditioned DCs on T cell polarization naïve T‐cell co‐cultured with HMOS‐conditioned DCs were stained with activation marker CD69 and intracellular Tbet and GATA3 as a Th1 and Th2 T‐cell marker, respectively. HMOS‐conditioned DCs showed no significant impact on the priming of CD69+Tbet+cells nor on CD69+GATA3+cellular polarization (Fig. 5B, C) as compared to control DCs. An increase in the percentage of Tbet+ cells was detected in the LPS‐stimulated co‐culture of DCs‐T‐cell compared to untreated control co‐culture (Fig. 5B right panel, *p* < 0.01). Interestingly, in the context of possibly excessive Th1 activation induced by LPS‐stimulated DC, HMOS effectively attenuated this effect in a concentration‐dependent manner (Fig. 5B right panel). No changes were detected in the percentage of GATA3+ expressing cells within the different DC‐T cell co‐cultures (Fig. 5C). No differences on IFN‐γ production by T‐cell were detected between HMOS‐conditioned DCs and untreated DCs, whereas a significant increase in IFN‐γ level was detected in the supernatant of CD4^+^ T‐cell primed with LPS‐conditioned DCs (Fig. 5E, *p* < 0.01). Interestingly, also a lower IFN‐γ level was measured in the supernatant of HMOS (5 mg/ml) + LPS‐DCs primed T‐cell compared to LPS‐DCs primed T‐cell (Fig. 5E, *p* < 0.05). In conjunction with the GATA3 expression also no differences were found in IL‐4 production between T‐cell primed by DCs with different treatment (Fig. 5F). Together, these findings indicate that HMOS *per se* don't seem to condition DCs to prime Th1 nor Th2 cells directly; HMOS were able to condition DCs to maintain the CD4^+^ T‐cell balance within the context of over‐activated Th1 responses induced by LPS stimulation.

### T cells primed by HMOS‐conditioned human moDCs suppress proliferation of responder CD4^+^ T cells

**Figure 6:**
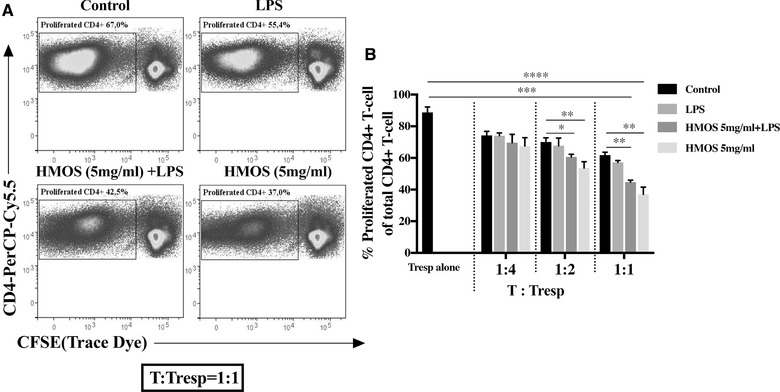
Suppressive capacity of HMOS‐conditioned human moDCs on activated responder CD4^+^ T‐cell proliferation. (A) Representative plots of T‐responder cell (Tresp) co‐cultured with CD4^+^T‐cell primed by control DCs, LPS treated DCs, HMOS (5 mg/ml) +LPS treated DCs, or HMOS (5 mg/ml) treated DCs at the ratio of 1:1. (B) The degree of Tresp proliferation after co‐culturing with CD4^+^T‐cell primed by different DCs. CD3/CD28 activated CFSE‐labelled responder CD4^+^T‐cell was co‐cultured with CD4^+^T‐cell primed by different DCs at ratio 4:1, 2:1, and 1:1 for 5 days. Proliferation of FITC‐positive cells was analyzed by flow cytometry and suppressive functionality was determined by comparing the proliferated CD4‐PerCP‐Cy5.5 positive T cell. Results are presented as mean ± SEM, (3 independent experiments (1‐2 donors/experiment). *p < 0.05, **p < 0.01, ***p < 0.01, ****p < 0.001, paired Student's t‐test.

Based on the finding that CD4^+^ T‐cell primed by HMOS‐conditioned DCs induce a regulatory phenotype, we tested the capacity of this primed CD4^+^ T cell pool to suppress proliferation of activated responder CD4^+^ T‐cell. CD4^+^ T‐cell obtained from the primary MLR were co‐cultured with CD3/CD28 activated CFSE‐labelled responder CD4^+^ T‐cell at different ratios (1:4, 1:2, 1:1) for 5 days. T‐cell primed by both 5 mg/mL HMOS‐ and 5 mg/mL HMOS+LPS‐conditioned DCs were capable to suppress the proliferation of activated responder CD4^+^ T‐cell significantly (Fig. 6B). The suppressive capacity was most pronounced in the 1:1 ratio co‐culture; the proliferation of activated responder CD4^+^ T‐cell was suppressed up to 43% in the presence of HMOS‐DCs primed T‐cell (Fig. 6B, *p* < 0.0001) and up to 39% in the presence of HMOS+LPS‐DCs primed T‐cell (Fig. 6B, *p* < 0.001), whereas only 28% and 22% suppression was measured in the presence of LPS‐DCs primed T‐cell and untreated‐DCs primed T‐cell, respectively, compared to the condition with only CD3/CD28 activated CFSE‐labelled responder CD4^+^ T‐cell. Overall, these data suggest that HMOS alone or in the presence of LPS, modulates the capacity of human moDCs to prime CD4^+^T‐cell with a suppressive capacity.

### HMOS induce regulatory DCs possibly through interaction with DC‐SIGN and TLR4 receptors

**Figure 7:**
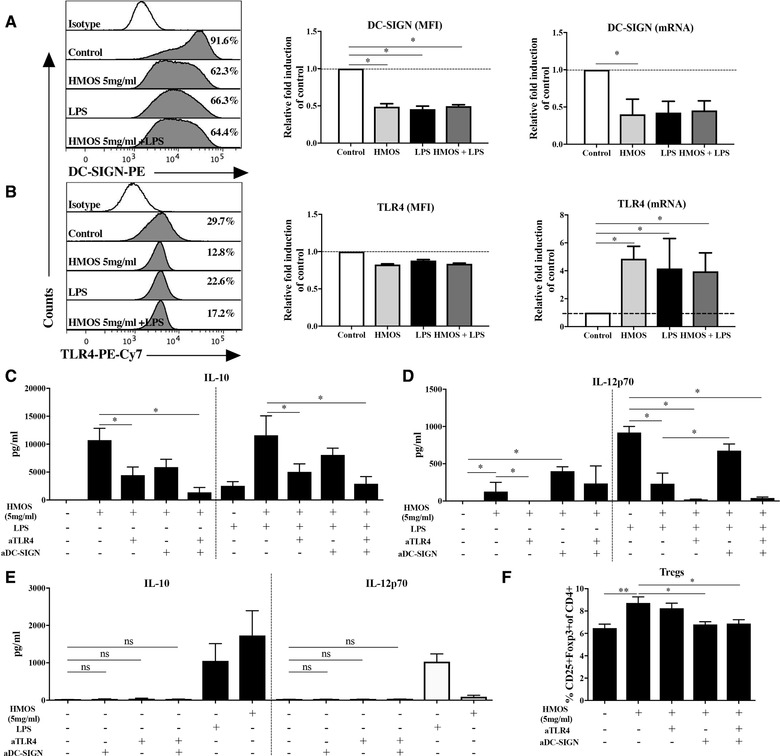
Effects of HMOS on TLR4 and DC‐SIGN expression and activity. (A) DC‐SIGN‐PE and (B) TLR4‐PE‐Cy7 expression on human monocyte‐derived immature DCs at protein and mRNA levels assessed by flow cytometry and QPCR assays, respectively. The effects of blockage of TLR4 or/and DC‐SIGN receptors on the production of (C,E) IL‐10, (D,E) IL‐12p70, and (F) Tregs generation. iDCs were pre‐incubated with anti‐TLR4 or/and DC‐SIGN antibodies for 2h before adding HMOS or/and LPS treatment, supernatant was collected at 24 h for the measurement of IL‐10 and IL‐12p70 by Elisa assays, DCs were collected and co‐cultured with naïve CD4^+^ T‐cell as described above. Results are presented as mean ± SEM, (4 independent experiments (1‐3 donors/experiment). *p < 0.05, **p < 0.01, paired Student's *t*‐test.

DC‐SIGN 29 and TLR4 30 are known pathogen recognition receptors regulating host microbe interaction. Therefore, we first compared expression levels of TLR4 and DC‐SIGN on the immature DCs (control) and conditioned DCs. Expression levels of DC‐SIGN (both at mRNA and protein levels) were observed to be lower on DCs cultured with HMOS (5 mg/mL), LPS, and the combination of HMOS (5 mg/mL) +LPS (Fig. 7A) as compared to non‐treated DCs. TLR4 expression at mRNA level was increased in the human moDCs treated by HMOS, LPS as well as the combination of HMOS+LPS (Fig. 7B) compared to the levels detected within untreated control DCs. However, at protein level, no changes were detected regarding TLR4 expression on human DCs by the different treatments (Fig. 7B). Next, we investigated the role of these two receptors in the IL‐10, IL‐12p70 production, and Treg induction capacity of DCs and the influence of HMOS in the presence or absence of LPS, by conducting receptor blocking assays. The addition of anti‐TLR4 antibody resulted in a reduction of IL‐10 levels detected in supernatants of HMOS‐induced human moDC as compared to control DCs (Fig. 7C, left panel, *p* < 0.05). Although IL‐10 levels were similar in co‐cultures when adding the anti‐DC‐SIGN antibody alone, a significant down‐regulation could be detected if the combination of anti‐DC‐SIGN and anti‐TLR4 antibodies were used (Fig. 7C left panel, *p* < 0.05). A similar pattern was observed in the presence of HMOS and LPS stimulation on DCs (Fig. 7C, right panel). Besides, the addition of anti‐TLR4 antibody resulted in no IL‐12p70 production, whereas DC‐SIGN blockage slightly increased IL‐12p70 production (Fig. 7D, left panel). In terms of the receptors involved in the effects of HMOS to suppress LPS‐induced IL‐12p70 released by Th1‐cell polarization of DCs, we found that blockage of DC‐SIGN receptor resulted in partial inability of HMOS to suppress LPS‐induced IL‐12p70 production (Fig. 7D, right panel, *p* < 0.05). The addition of anti‐TLR4 alone or combined with anti‐DC‐SIGN antibody led to a complete suppression on IL‐12p70 release (Fig. 7D, right panel, *p* < 0.05). The addition of blocking antibodies did not show any significant effects on the production of IL‐10 and IL‐12p70 compared to negative control treatment (Fig. 7E). In addition, within the DC‐T‐cell co‐culture experiment a reduced percentage of HMOS‐induced Tregs generation by adding anti‐DC‐SIGN as well as the combination of anti‐DC‐SIGN and ‐TLR4 antibodies was detected (Fig. 7F, *p* < 0.05 and *p* < 0.05, respectively). Whereas no effect was observed in the generation of Tregs induced by HMOS‐conditioned DCs by the addition of anti‐TLR4 antibody alone (Fig. 7F). These data suggest that both DC‐SIGN and TLR4 receptors signaling are involved in HMOS conditioned DC induced IL‐10 production, whereas DC‐SIGN signaling seems to be primarily involved in generation of Tregs. These findings together indicate that DC‐SIGN signaling indeed contributes to the suppressive capacity of HMOS on LPS‐induced activation in human DCs. However, it is unclear whether binding of specific HMOS with TLR4 impacts the inhibition on LPS‐induced activation since addition of anti‐TLR4 antibody blocks the binding of both HMOS and LPS with TLR4.

## Discussion

During the first months of life, breastfeeding plays an essential role in programming the immune system, in part by modulation of the microbiota composition 31, 32. The developing and rapidly changing microbial community will constantly interact with the immune system. More importantly, breastfeeding also directly influences the immune system by providing the infants with immune modulatory compounds such as HMOS. Several HMOS structures have been documented not to be digested by GI enzymes but to be absorbed into the systemic circulation in both breastfed infants 14 and rats orally administrated with HMOS 15, suggesting that immune cells such as DCs are likely to be exposed to HMOS throughout the body. The immune modulatory effects of prebiotic oligosaccharides mixture 22, as well as specific HMOS 3’FL and 6’SL 24 on human moDCs have been described by previous studies. However, the effects of the authentic (i.e. complete) HMOS mixture on the human DC phenotype and function have not been reported before. In the present study, we show for the first time that HMOS modulate human moDCs in a microbiota‐independent way. The unique mixture of HMOS, isolated from mature human milk, induces a semi‐maturation state and suppresses LPS‐induced immune activation of human moDCs. These HMOS‐conditioned DCs are then able to modulate T‐cell responses in which both TLR4 as well as DC‐SIGN seem to play a role, suggesting a key role of HMOS in early life immune development.

A recent study indicates that tolerogenic DCs induced by one specific HMOS 3’SL is attributed to LPS contamination 24, indicating the necessity of evaluating potential LPS contamination in HMOS preparations. A low level of endotoxins was detected in the HMOS preparation (data not shown), however upon removal of potential LPS contamination by the addition of PB, the observed immune modulatory effects of HMOS on human moDCs were not significantly influenced. In addition, HMOS‐conditioned DCs induced a remarkably different pattern of membrane co‐stimulatory markers expression, cytokine production and DC‐T‐cell interactions than LPS‐stimulated DCs, supporting the notification that HMOS do exert immune modulatory effects on human DCs. On the other hand, since microbial components were detected within the HMOS preparation, we cannot rule out the possibility that they play an essential role in combination with HMOS to steer the moDC generation and maturation. Which in itself makes sense, because upon birth all infants will be exposed to a huge amount of different microbial components while forming a stable microbiome. This even suggests that HMOS might exert their effects specifically in the presence of microbial and or pathogenic antigens 33. In this regard it is also known that both microbial start in early life (i.e. c‐section versus natural delivery) as well as the provision of human milk is essential for development of infant's immune system 8.

Studies have demonstrated or suggested direct interactions of specific HMOS or prebiotic oligosaccharides with murine and/or human DCs 23, 24, 26. HMOS are potential gut microbiota modulators which effectively stimulate the growth of beneficial bacteria such as Bifidobacteria, and may subsequently increase bacterial metabolites such as SCFAs production as result of fermenting HMOS 34. Both the microbiome alterations 35, 36 and SCFAs 37, 38 are known to effectively influence phenotype and function of human DCs. In addition to these features, we show that HMOS concentration‐dependently induce a semi‐maturation of human moDCs. The up‐regulation of cytokines such as IL‐10 and IL‐27 indicates a direct immune regulatory property of HMOS on human moDCs. This is similar to the effects detected by scGOS/lcFOS on human moDCs 22. Proper activation of innate immune cells is essential for immune education in early life, therefore activation of DCs by HMOS may be essential for immune development in neonates 39. On the other hand, over activation of immune responses may cause autoimmune diseases and excessive inflammation 40. HMOS suppress LPS‐induced expression of DCs surface markers and reduce LPS‐mediated (pro‐) inflammatory cytokines IL‐12p70 (IL‐12p40), IL‐6, TNF‐α gene expression and protein release. These findings are in line with the previously reported feature of specific HMOS 2’FL, which effectively attenuated LPS‐mediated inflammation in human enterocytes 41. However, the impact of the total HMOS mixture or individual HMOS on LPS‐induced biological activity on human DCs have not been described previously and show a critical co‐evolution of both the prebiotic oligosaccharides in conjunction with the microbiome. It's worth mentioning that the moDCs generated in current study do not have a clear separation between CD11C_high, CD11C_inter and CD11C_low populations and therefore might also contain minor population of other immune cells (most likely Macrophages), which might also contribute to the observed immune modulatory effects of HMOS. This opens new perspectives for investigating the direct interactions between HMOS and other immune cells such as macrophages in the future.

Semi‐mature DCs, defined by MHC‐II^high^, costimulation^high^, pro‐inflammatory cytokines^low^, are potent inducers of IL‐10‐producing Treg 42. In addition, this type of DCs promote Treg differentiation and limit effector T‐cell in the periphery by production of IL‐10, IL‐27, and TGF‐β 16. Our data regarding HMOS induced DC phenotype and cytokine profile indicating a promotion of Tregs accompanied by higher IL‐10 release. In addition, the HMOS+LPS‐conditioned DCs displayed similar effects on both generation of Tregs and IL‐10 release, supporting the notification of HMOS condition human moDCs with the capacity and stability to induce immune resilience. Our observation is in accordance with earlier in vitro study showing that non‐digestible oligosaccharides‐induced semi‐mature and IL‐10 producing DCs promoted generation of Tregs 22. Furthermore, the up‐regulated levels of chemokine receptors CXCR3 and CCR7 suggest that HMOS might enable DCs even to migrate to lymphoid organs and inflammatory lesions, respectively. Consistent with previous studies 28, we found that LPS stimulation was essential in order to enhance this capacity induced by HMOS. However, further studies should be conducted to prove this capacity in vivo as well as to understand the full range of detailed host‐microbe interaction.

Dietary intervention with HMOS in vivo has shown to significantly reduce diabetes incidence, possibly through reducing the induction of diabetogenic cytokines such as IL‐17 and IFN‐γ in a NOD‐mice model 43. On the other hand, in line with known effects of scGOS/lcFOS, we have recently demonstrated that specific HMOS, namely 2’FL, can effectively enhance vaccine‐specific immune responses by inducing Th1 type of immunity including IFN‐γ responses within an influenza vaccination mouse model 44. In this regard, HMOS induced an anti‐inflammatory cytokine profile of DCs that promotes IL‐10 producing Tregs generation in a steady state, suggesting regulatory function of HMOS in vivo.

Mechanistically, HMOS recognition by human moDC may be regulated by receptors including but not limited to DC‐SIGN and TLR4. DC‐SIGN is involved in the induction of IL‐10 and Tregs by DCs, and TLR4 signaling is involved in IL‐10 production by DCs as well. Although we have observed that blockage of TLR4 abrogated IL‐12p70 production by DCs treated with the combination of HMOS and LPS, it is difficult to conclude if it is the HMOS‐TLR4 interaction that influenced LPS‐induced IL‐12p70 production since addition of TLR4 blocking antibody might block the binding of both specific HMO(S) and LPS with TLR4. Previous studies regarding the potent receptors involved in specific oligosaccharide of the total HMOS mixture sensing by DCs indicated involvement of C‐type lectin DC‐SIGN in 2’FL‐induced immune regulation 26, TLR4 as for 3’SL‐induced immune regulation 23 and Siglecs as a receptor of 3’SL‐, 6’SL‐induced immune regulation 26. However, to the best of our knowledge, we now for the first time show that both DC‐SIGN and TLR4 receptors are equally important in driving immune regulatory properties of HMOS mixture of human DCs. One possible explanation is that different individual oligosaccharides existing within the HMOS mixture have different affinities for specific receptors. Based on existing evidence 23, it can be speculated that specific HMOS, such as 3’SL may act as a competitor of LPS for binding to TLR4 receptor in human moDCs, thereby suppressing LPS‐induced activation of DCs. However, further studies are necessary to identify which individual HMOS bind to specific receptors, as well as which pathways of DC‐SIGN and TLR4 signaling would be involved in the HMOS‐induced regulatory effects.

In summary, for the first time we showed that the authentic mixture of HMOS isolated from human milk equips human moDCs with a regulatory phenotype and function, and subsequently induces Treg expansion. In addition, we demonstrate that HMOS effectively suppress LPS‐induced maturation of human moDCs, thereby inhibiting LPS‐induced pro‐inflammatory responses. The regulatory phenotype of HMOS‐conditioned DCs is dependent on interactions of HMOS with TLR4 and DC‐SIGN receptors on human moDCs. These findings open further perspectives regarding the potential of HMOS to beneficially regulate the immune responses and shape the inflammatory response in a microbiota‐independent way through DCs in early life.

## Materials and methods

### Human milk oligosaccharides

HMOS were isolated and purified from pooled mature human milk samples donated by healthy donors as described previously 43. This authentic HMOS mixture consists of both long‐chain as well as short‐chain structures, (in approx. 9/1 ratio), including neutral core oligosaccharides with fucosylation and/or sialylation ion, neutral and acidic oligosaccharides, in total 84% HMOS and 16% lactose. The structural distribution used in the current study is shown in Supporting Information Fig. 1. Structure analysis was performed using gel permeation chromatography (GPC), as previously described 45, 46. The characterization was performed by either CGE‐LIF, LC or mass spec or a combination of both as described elsewhere 47.

### Generation, stimulation and analysis of human monocyte‐derived dendritic cells

To generate moDCs, human PBMCs were isolated from buffy coats by means of density gradient centrifugation using LeucoSep tubes (Greiner). CD14^+^ monocytes were isolated by CD14 MicroBeads (Miltenyi Biotec) and cultured in RPMI‐1640 with L‐Glutamine supplemented with 10% fetal calf serum (Bodinco), 100U/mL penicillin/streptomycin, 10 mM HEPES, 1 mM sodium pyruvate and Eagles minimum essential medium (MEM) non‐essential amino acids (all from Gibco Life Technologies) in the presence of 500 U/mL IL‐4 and 800 U/mL GM‐CSF (both ProSpec). The monocytes were cultured in a 5% CO_2_ incubator at 37°C. Immature moDCs were harvested at day 6 and seeded into a 12‐wells plate (Greiner) at a density of 5 × 10^5^ cells/well in culture medium without IL‐4 and GM‐CSF. Immature DCs were either left unstimulated (iDCs, control) or treated with HMOS (0.8, 2, 5 mg/mL) in the presence or absence of 100 ng/mL ultrapure LPS (*E. coli* 026: B6 strain, eBioscience, the Netherlands). After 24 h of stimulation, DCs were collected and analyzed by flow cytometry (FACS Canto II) according to the recently published guidelines 48. Cells were first stained for viability using Fixable viability dye eFluor 780 (eBioscience, the Netherlands), followed by extracellular staining of surface markers CD14 (APC), CD80 (PE), CD86 (PE), CD40 (PE‐Cy7), HLA‐DR (PerCp Cy5.5) and PD‐L1 (PE‐Cy7), OX40L (PerCP‐Cy5.5), CXCR3 (FITC), CCR7 (PerCP‐Cy5.5), DC‐SIGN (PE), TLR4 (PE‐Cy7). Furthermore, supernatant was collected and stored at −80°C to measure cytokine levels of IL‐4, IL‐6, IL‐10, IL12p70, IL‐27, and TNF‐α by Elisa according to the manufacturer's protocol (all were purchased from eBioscience, the Netherlands).

### Allogenic stimulation assay (ASA) of naïve CD4^+^ T‐cell

To assess functionality, moDCs (24h‐stimulated as described above) were co‐cultured with allogenic naïve CD4^+^ T‐cell for 6–7 days in a 96‐well U‐bottom plate (Greiner) at a 1:10 ratio. The naïve CD4^+^ T cells were purified from PBMCs by MACS (Naïve CD4^+^ T‐cell isolation kit II, Miltenyi Biotec) and purity was around 98% as assessed by flow cytometry after isolation. After 6–7 days of co‐culture, supernatant was collected and stored at ‐80°C until measurement of cytokine levels (IFN‐γ, IL‐10, and IL‐4) by ELISA (all kits from eBioscience, the Netherlands). Furthermore, the polarization of T cells was analyzed using flow cytometry. Cells were first stained for viability using FVD (APC‐Cy7), followed by extracellular staining of CD4 (PerCp‐Cy5.5), CD25 (FITC) and CD69 (FITC). Cells were fixed and permeabilized using the Foxp3 fixation/permeabilization kit (eBioscience, the Netherlands). Subsequently, cells were stained for intracellular markers FoxP3 (PE‐Cy7), GATA3 (PE) and Tbet (PE‐Cy7). All antibodies were purchased from eBioscience (The Netherlands).

### mRNA isolation and quantitative real‐time PCR

mRNA was extracted from snap‐frozen 16h‐stimulated moDCs using a NucleoSpin® RNA Plus kit (Macherey‐Nagel, Germany) in combination with the rDNAse set (Macherey‐Nagel, Germany) to remove contaminating DNA. cDNA was synthesized using an iScript™ advanced kit (Bio‐Rad, USA) according to the manufacturer's protocol in the PTC‐100 Programmable Thermal Controller (MJ research, PTC‐100). cDNA and mRNA samples were stored at ‐80°C. Quantitative analysis was conducted on a CFX96 Real‐Time C1000 Thermal Cycler detection system with the use of an IQ™ SYBR® Green Supermix, according to manufacturer's protocol (both from Bio‐Rad). Primers for the genes of interest are listed in Table 1. Primers (produced by Biolegend, USA) were designed with Primerblast (NCBI, USA) and Beacon Designer (Premier Biosoft International, USA). Primers were validated based on the efficiency and R^2^. Primer specificity was tested by running the products on a 2% agarose gel (70 minutes; 110V). The expression of the genes of interest was determined using 12.5ng cDNA and SYBR green (Bio‐rad, USA). The RT‐PCR protocol consists of 3 min at 95°C followed by 10 s at 95°C and 30 s at the optimal primer annealing temperature as determined beforehand. The samples were measured in duplo and mean Ct values were used to determine the relative expression using the Pflaff method and β‐actin as a reference gene.

**Table 1:** Sequences of specific primers for detected genes

Gene ID	Forward primer sequence	Reverse primer sequence
CD80	GCAGGGAACATCACCATCCA	ACGTGGATAACACCTGAACAGA
CD86	CACGGATGAGTGGGGTCATT	AAGTTAGCAGAGAGCAGGAAGG
CD40	TGGTGAGTCCTGGACAATGG	ACCTTTTTGATAAAGACCAGCACCA
HLA‐DRA	AGCACTGGGAGTTTGATGCT	GCTTTTGCGCAATCCCTTGA
HLA‐DRB	CCCTGAGTGAGACTTGCCTG	GAAACGTGGTCGGGTGTCC
PD‐L1	GTGAAAGTCAATGCCCCATACA	TGTCCAGATGACTTCGGCCT
IL‐10	TACGGCGCTGTCATCGATTT	TAGAGTCGCCACCCTGATGT
IL‐27p28	GAGCAGCTCCCTGATGTTTC	AGCTGCATCCTCTCCATGTT
IL‐12p40	AGGGACATCATCAAACCTGACC	GCTGAGGTCTTGTCCGTGAA
IL‐6	CAACCCCCAATAAATATAGGACTGG	GGACCGAAGGCGCTTGTG
TGF‐β	CCGTGGAGGGGAAATTGAG	TGAACCCGTTGATGTCCACTT
TNF‐α	CCTGCTGCACTTTGGAGTGA	GAGGGTTTGCTACAACATGGG
CCR7	TGTGGTTTTACCGCCCAGAG	TGACACAGGCATACCTGGAAAA
CXCR3	TGGTCCTTGAGGTGAGTG	GCACGAGTCACTCTCGTTTT
β‐actin	AGCACAGAGCCTCGCCTTT	CATCACGCCCTGGTGCCT

### Functional T‐cell suppression assay

To analyze the suppressive capacity of the different DC‐induced T‐cell, cells were harvested from the ASA 96‐well plate on day 7, thoroughly washed and co‐incubated with freshly isolated allogenic responder CD4^+^T‐cell at a ratio of 1:1, 1:2, and 1:4. To track the proliferation of responder T‐cell in the suppression assay, responder cells were pre‐labeled with CFSE (Thermo Fisher Scientific, The Netherlands) according to the manufacturers’ instructions, at a final concentration of 1uM. Dynabeads® human T‐Activator CD3/CD28 (Thermo Fisher Scientific, The Netherlands) were added to activate the responder T‐cell. After 5 days, the mix of ASA cells and CFSE labeled responder CD4^+^ T‐cell was stained with Fixable viability dye eFluor 780 and CD4 APC (both eBioscience, the Netherlands). Suppressive capacity was determined by setting gates on proliferated CD4^+^ T‐cell and comparing the percentage of proliferated cells of different conditions.

### DC‐SIGN and TLR4 blocking assays

Immature human moDCs were pre‐incubated with anti‐TLR4 and anti‐DC‐SIGN (both are purchased from Abcam, the Netherlands) for 2 h before adding HMOS and/or LPS. Subsequently, cells were cultured for 24 h to collect supernatant for the measurement of IL‐10 and IL‐12p70. Treated DCs were thoroughly washed and co‐cultured with isolated purified naïve CD4^+^ T‐cell for the measurement of Treg generation by flow cytometry.

### Ethics statement

For this study, buffy coats of healthy donors were purchased from Sanquin (Amsterdam) as a source of peripheral blood mononuclear cells (PBMCs). The human milk sample collection, storage and use were approved and carried out in accordance to European guidelines and regulations and described in 43. All healthy donors (tested negative for Hepatitis and HIV) provided informed consent to use the voluntary provided human milk sample for Research Purpose in accordance with the Declaration of Helsinki. As such, no further ethical approval was required for these studies.

### Statistics

Statistical analysis was performed using GraphPad Prism 7.0 (GraphPad Software, La Jolla, CA, USA). Paired Student's *t*‐test was used to assess differences between two conditions. *p*<0.05 was considered as significant. Data were represented as mean ± SEM.

## Author contributions

LX, BL, GF, designed the experiments; LX, WW, RS, CM, NK, BB, SO performed experimental procedures; LX performed data collection, analysis and drafted the manuscript; JG, GF and BL supervised the program; BS made specific contributions to the program regarding the human milk oligosaccharides. All authors listed have made significant contribution to writing the manuscript and approved it for publication.

## Conflict of interest

None of the authors have a competing financial interest in relation to the presented work; JG is head of the Division of Pharmacology, Utrecht Institute for Pharmaceutical Sciences, Faculty of Science at the Utrecht University, and partly employed by Nutricia Research. NK, BS, BB, SO, and BL are employed by Nutricia Research. BL, as indicated by the affiliations, is leading a strategic alliance between University Medical Centre Utrecht/Wilhelmina Children's Hospital and Nutricia Research.

**Abbreviations**
2’FL: 2’‐fucosyllactose
3’SL: 3’‐sialyllactose
6’SL: 6’‐sialyllactose
ASA: Allogenic Stimulation Assay
DCs: dendritic cells
DC‐SIGN: DC‐specific intercellular adhesion molecule‐3‐grabbing non‐integrin
lcFOS: long‐chain fructo‐oligosaccharides
Foxp3: forkhead box protein 3
FUT 2: fucosyltransferase 2
HMOS: human milk oligosaccharides
iDC: immature DCs
MFI: median fluorescence intensity
LNFP‐I: Lacto‐N‐Fucopentaose I
MLN: mesenteric lymph node
MLR: allogenic mixed lymphocyte reaction
moDCs: monocytes‐derived DCs
PBMC: peripheral blood mononuclear cells
PB: polymyxin B
Siglecs: sialic acid binding immunoglobulin‐like lectins
SCFAs: short chain fatty acids
scGOS: short‐chain galacto‐oligosaccharides
Th: T‐helper
Tregs: regulatory T‐cell
TLR4: toll‐like receptor 4

## Supporting information

Supporting Information
