# Supplementary material for: Human milk oligosaccharides promote immune tolerance via direct interactions with human dendritic cells
Source: Eur J Immunol. 2019 Apr 4;49(7):1001–14. doi: 10.1002/eji.201847971 (PMC6619030; doi:10.1002/eji.201847971)
Supplement: Supplementary file 1 — Supporting Information [file EJI-49-1001-s001.pdf]

European Journal of Immunology

Supporting Information

for

DOI 10.1002/eji.201847971

Ling Xiao, Wouter RPH van De Worp, Roderick Stassen, Celine van Maastrigt, Nienke Kettelarij, Bernd Stahl, Bernadet Blijenberg, Saskia A. Overbeek, Gert Folkerts, Johan Garssen and Belinda van't Land

Human milk oligosaccharides promote immune tolerance via direct interactions with human dendritic cells

© 2019 The Authors. *European Journal of Immunology* published by WILEY-VCH Verlag GmbH & Co. KGaA, Weinheim.

Supporting Information

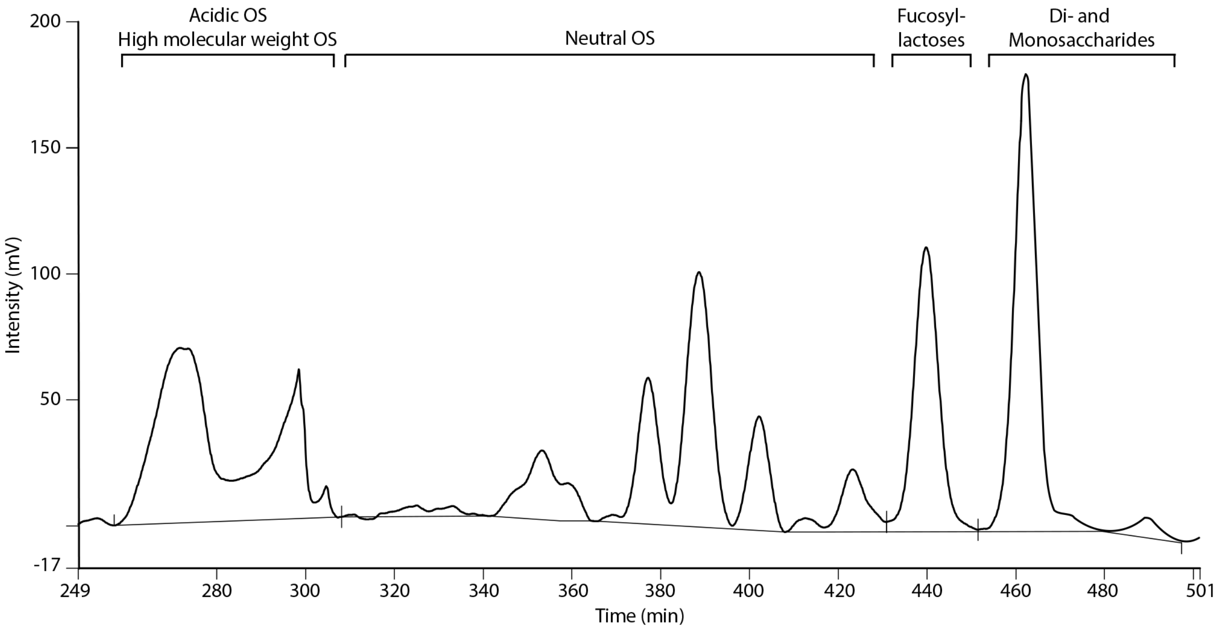

Peak name	Area (mV*min)	Relative area (%)	Glycosidic structure
Acidic OS<br>High molecular weight OS	1490.99	28.60	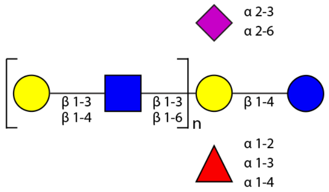
Neutral OS	1790.45	34.35	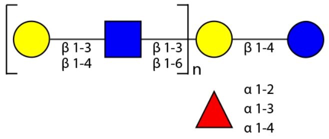
Fucosyllactoses	745.688	14.3	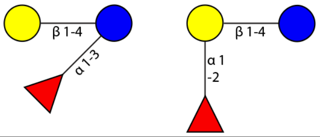
Di- and<br>Monosaccharides	1185.98	22.75	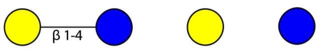

Supplementary Figure 1. Distribution pattern of HMOS mixture by GPC. The HMOS mixture was injected in a 2 times 5 x 110 cm Kronlab ECO50 column, packed with Toyopearl HW 40 (S) (TOSOH BIOSCIENCE). The chromatogram was obtained by refractive index detection.

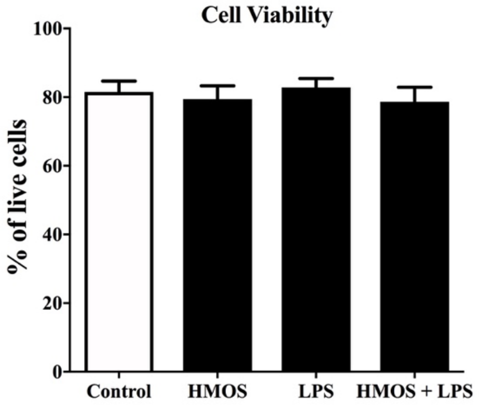

Supplementary Figure 2. Cell viability of DCs treated by different conditions. Neither LPS nor HMOS (5 mg/ml) did affect the cell viability compared to control. Mean± SEM is shown, N=6, Mann-Whitney test, no significant difference was observed between groups.

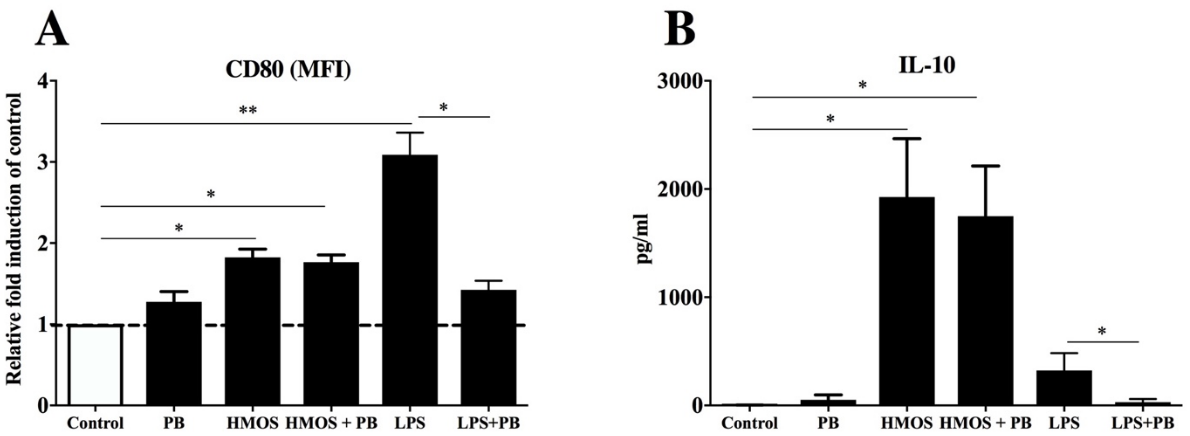

Supplementary Figure 3. Removal of potential LPS contaminations by polymyxin B does not change the effects of HMOS. HMOS (5 mg/ml) or LPS solution was incubated with 10μg/ml polymyxin B for 1h before treatment with DCs, after 24h, **(A)** CD80 expression and **(B)** IL-10 levels in the supernatant were analyzed by flow cytometry and Elisa assay, respectively. Mean± SEM is shown, N=3, Mann-Whitney test, *p<0.05, **p<0.01.

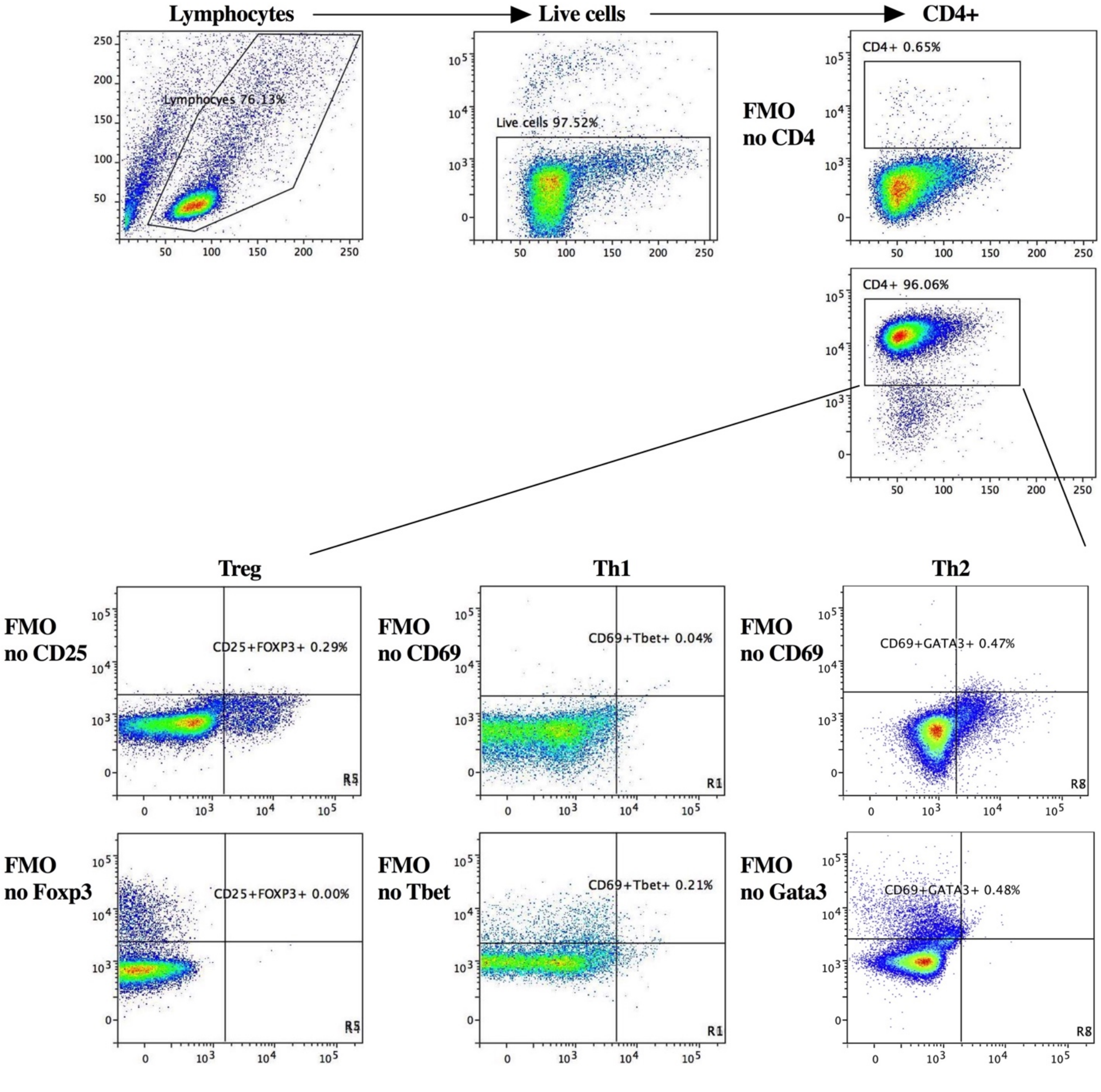

Supplementary Figure 4. Gating strategy T-cell. After 6 days of co-culture, cells were collected and analyzed by flow cytometry. Cells were first stained for viability using FVD (APC-Cy7), followed by extracellular staining of CD4 (PerCp-Cy5.5), CD25 (FITC) and CD69 (FITC). After fixation and permeabilization, cells were stained for intracellular markers FoxP3 (PE-Cy7), GATA3 (PE) and Tbet (PE-Cy7). Flow cytometry analysis software Flowlogic 7 (Inivai Technologies) was used to analyze the data. In our gating strategy, first, the singlets were selected. Next the lymphocytes were selected based on FSC and SSC. Out of the lymphocyte gate the alive cell population was selected. CD4+ cells were obtained out the gate of alive cells, using FMO control. CD25+ Foxp3+, CD69+ GATA3+ and CD69+ Tbet+ cells were derived out the gate of CD4+ cells, using FMO controls.
